# Supplementary material for: Long-term outcomes of peripheral arterial disease patients with significant coronary artery disease undergoing percutaneous coronary intervention
Source: PLoS One. 2021 May 19;16(5):e0251542. doi: 10.1371/journal.pone.0251542 (PMC8133421; doi:10.1371/journal.pone.0251542)
Supplement: Attachment — Submitted filename: Comments to Authors(Dr wang).doc [file pone.0251542.s001.doc]

**Comments to Authors:**

The authors provide an interesting and potential important manuscript describing"Long-term Outcomes of Peripheral Arterial Disease Patients with Significant Coronary Artery Disease underwent Percutaneous Coronary Intervention" The main issues concerning this paper are those concerning the relationship between PAD and CAD. The previous study had figured out that 54%-69% of CAD patients with PAD, but a limited paper had researched that the percent of CAD in PAD patients.This study was designed to observe CAD in patients with PAD, and conclude that patients with PAD often have CAD disease.

There are some weak points that need to be addressed by the authors.

Major

1. In all patient analysis, the CAD patients showed a significantly higher prevalence of DM as compared with non-CAD subjects (*P* < 0.01). However, in age-matched analysis, there was no significant difference between two groups. The authors explain it.
2. As shown in Table 4, in all patients, total death (five-year outcomes) was higher in CAD group than in non-CAD group without significant difference. In contrast, in age matched analysis, it was higher non-CAD group than that of CDA patients. Why?
3. Discussion sections were disorganized, the authors did not clearly figured out their own findings and did not derive clinical implication in each section based on your own and previous observations..
