# Supplementary material for: Long-term outcomes of peripheral arterial disease patients with significant coronary artery disease undergoing percutaneous coronary intervention
Source: PLoS One. 2021 May 19;16(5):e0251542. doi: 10.1371/journal.pone.0251542 (PMC8133421; doi:10.1371/journal.pone.0251542)
Supplement: Attachment — Submitted filename: Pone-PTA-CAD-Rebuttal-Rha.doc [file pone.0251542.s002.doc]

PONE-D-19-21312

Long-term Outcomes of Peripheral Arterial Disease Patients with Significant Coronary Artery Disease undergoing Percutaneous Coronary Intervention

Dear editor:

Thank you so much for your e-mail sent to us on November 29 2019. We really appreciate the great opportunity you have kindly given to us to resubmit our work!! We really appreciate your excellent advice which has helped us a lot to improve the quality of our manuscript. According to your comments, we have amended the relevant parts in the manuscript. All of your questions were answered below. In the process of revising the manuscript, Jin-Seok Kim was added as an author, and he was fully involved in the revision of the study. All authors have approved Dr. Kim's inclusion as an author.

Yours sincerely,

***Correspondence to:**

Seung-Woon Rha, MD, PhD, E‐mail: swrha617@yahoo.co.kr (S-WR)

Byoung Geol Choi, PhD, E‐mail: trv940@korea.ac.kr (BGC)

Journal Requirements:

When submitting your revision, we need you to address these additional requirements.

1) Please ensure that your manuscript meets PLOS ONE's style requirements, including those for file naming. The PLOS ONE style templates can be found at

http://www.journals.plos.org/plosone/s/file?id=wjVg/PLOSOne_formatting_sample_main_body.pdf and http://www.journals.plos.org/plosone/s/file?id=ba62/PLOSOne_formatting_sample_title_authors_affiliations.pdf

**Response: Thank you so much for your careful review. We reflected this in the revised manuscript.**

2) During your revisions, please note that a simple title correction is required: "Long-term Outcomes of Peripheral Arterial Disease Patients with Significant Coronary Artery Disease *undergoing* Percutaneous Coronary Intervention". Please ensure this is updated in the manuscript file and the online submission information.

**Response: Thank you so much for your careful review and comment. We reflected this in the title of the revised manuscript as following as “Long-term Outcomes of Peripheral Arterial Disease Patients with Significant Coronary Artery Disease undergoing Percutaneous Coronary Intervention”**

3) Thank you for stating the following financial disclosure:

[The authors have no financial conflicts of interest relevant to the manuscript to

disclose.].

Please provide an amended Funding Statement that declares *all* the funding or sources of support received during this specific study (whether external or internal to your organization) as detailed online in our guide for authors at http://journals.plos.org/plosone/s/submit-now.

Please state what role the funders took in the study. If any authors received a salary from any of your funders, please state which authors and which funder. If the funders had no role, please state: "The funders had no role in study design, data collection and analysis, decision to publish, or preparation of the manuscript."

Please include your amended statements within your cover letter; we will change the online submission form on your behalf.

**Response: Thank you so much for your careful review and comment. We reflected this in the revised manuscript (line 18 on page 1 to line 22) and the cover letter.**

4) In your Data Availability statement, you have not specified where the minimal data set underlying the results described in your manuscript can be found. PLOS defines a study's minimal data set as the underlying data used to reach the conclusions drawn in the manuscript and any additional data required to replicate the reported study findings in their entirety. All PLOS journals require that the minimal data set be made fully available. For more information about our data policy, please see http://journals.plos.org/plosone/s/data-availability.

Upon re-submitting your revised manuscript, please upload your study’s minimal underlying data set as either Supporting Information files or to a stable, public repository and include the relevant URLs, DOIs, or accession numbers within your revised cover letter. For a list of acceptable repositories, please see http://journals.plos.org/plosone/s/data-availability#loc-recommended-repositories. Any potentially identifying patient information must be fully anonymized.

Important: If there are ethical or legal restrictions to sharing your data publicly, please explain these restrictions in detail. Please see our guidelines for more information on what we consider unacceptable restrictions to publicly sharing data: http://journals.plos.org/plosone/s/data-availability#loc-unacceptable-data-access-restrictions. Note that it is not acceptable for the authors to be the sole named individuals responsible for ensuring data access.

We will update your Data Availability statement to reflect the information you provide in your cover letter.

**Response: Thank you so much for your careful review and comment. We reflected this in the revised manuscript (line 21 on page 1).**

**Dear reviewer:**

**Thank you so much for your very insightful and serious comments for our manuscript! We really appreciate your excellent advice which has helped us a lot in improving our manuscript. According to your comments, we have amended the relevant parts in the manuscript. Some of your questions were answered below.**

**Yours sincerely,**

***Correspondence to:**

Seung-Woon Rha, MD, PhD, E‐mail: swrha617@yahoo.co.kr (S-WR)

Byoung Geol Choi, PhD, E‐mail: trv940@korea.ac.kr (BGC)

Reviewer #1: The main finding of this study is that routine coronary angiography and subsequent percutaneous intervention for significant coronary artery disease in patients with symptomatic peripheral arterial disease is safe and resulted in similar long-term survival to those who did not have coronary artery disease.

1 The correlation of coronary and peripheral arterial disease is well known and treatment strategies have been discussed and examined. What therefore is the novelty in this study of its conclusions

**Response: Thank you so much for your careful review and important comment. As you understand, the treatment of PAD and CAD is very important for improving patient’s survival and quality of life. However, in patients undergoing PTA, routine evaluation of the presence of significant coronary artery disease may be controversial. In real world clinical practice, physicians who are performing endovascular intervention commonly only focusing on the extremity target lesion intervention without concerning of coronary artery disease evaluation and management, especially in particular specialties such as vascular surgeon or interventional radiologists. Thus, main intention of this report is to provoke all the endovascular intervention specialties should check patient’s co-existing significant CAD and to have an idea to safely manage the CAD together to prevent future cardiovascular events. Routine CAD checkup is not commonly widely accepted in daily clinical practice, especially in terms of cost-effectiveness but this should be changed according to our novel data.**

**Therefore, we added the following in the second paragraph with a conclusion in the last paragraph of the discussion part (line 1 on page 10 to line 4)**

2 How does a single center study that performed angiography on each patients help
a. Would it not have been more meaningful to compare those with symptoms who did and did not undergo angiography
**Response: Thank you so much for your careful review and important comment. Korea University Guro Hospital (KUGH), which conducted this study, is a university hospital for training, and is a large-scale center that performs many CAG, PCI, and PTAs every year. Data are collected by a trained research coordinator using a standardized case report form. And professional researchers analyze it consistently. It may be smaller than a multicenter study, but it can collect and analyze data more efficiently and accurately. Our study is a comparative study of the CAD group and the non-CAD group who received appropriate treatment through coronary artery evaluation in patients undergoing PTA. Therefore, the group without CAG was excluded. Main purpose of this study is to show whether the concomitant optimal revascularization of significant CAD regardless of patient’s symptom in PAD patients undergoing PTA could reduce the cardiovascular event risk compared with PAD patients without significant CAD. Selective CAD evaluation according to ischemic symptoms in PAD patients undergoing PTA is not a suitable and safe strategy because many of them are elderly, diabetics and limited ambulation to provoke ischemic chest pain or dyspnea due to diabetic foot wound. Thus, selective symptom driven CAD evaluation cannot be ideally accepted in context of ‘pan-atherosclerosis’ of PAD patients. We will consider a comparative study in symptomatic PAD patients between CAG and non-CAG groups in future studies.**

3 What new conclusions were learned not seen in ref, 8 the authors 1-year experience with this group

**Response: Thank you so much for your careful review and important comment. The results of our previous study, which observed the results for one year, the CAD group that received optimal treatment through routine coronary artery evaluation showed similar clinical results than the non-CAD group in CLI patients received PTA [8]. Similarly, in the present observation of long-term clinical results of 5-year in patients undergoing PTA, the clinical results of the CAD and non-CAD groups were similar. The strategy for CAD evaluation and treatment in PTA patients seems to be a safe and effective strategy not only for better short-term outcomes but also durable long-term outcomes. We wanted to show the durable long-term results from this routine CAD work up and management strategy in PAD patients undergoing PTA. We added this to the discussion part (line 20 on page 9 to line 14 on page 10).** 

4 Was a power calculation performed to determine if 160 matched patients could indeed detect a meaningful difference
**Response: Thank you so much for your careful review and important comment. This study was designed as a prospective registration study and retrospective analysis was performed. Therefore, the sample size after PSM may not be sufficient, which may be a limitation of the study. We added this to the limitations of the discussion part (line 17 on page 10 to line 19) as following as “Second, In the PSM analysis, a total of 320 patients from 160 pairs in both groups were analyzed. This sample size may be insufficient to produce results. Our study is a registered observational study and may be a limitation of analysis.”**

**Dear reviewer:**

**Thank you so much for your very insightful and serious comments for our manuscript! We really appreciate your excellent advice which has helped us a lot in improving our manuscript. According to your comments, we have amended the relevant parts in the manuscript. Some of your questions were answered below.**

**Yours sincerely,**

***Correspondence to:**

Seung-Woon Rha, MD, PhD, E‐mail: swrha617@yahoo.co.kr (S-WR)

Byoung Geol Choi, PhD, E‐mail: trv940@korea.ac.kr (BGC)

Reviewer #2: Comments to Authors:

The authors provide an interesting and potential important manuscript describing "Long-term Outcomes of Peripheral Arterial Disease Patients with Significant Coronary Artery Disease underwent Percutaneous Coronary Intervention" The main issues concerning this paper are those concerning the relationship between PAD and CAD. The previous study had figured out that 54%-69% of CAD patients with PAD, but a limited paper had researched that the percent of CAD in PAD patients. This study was designed to observe CAD in patients with PAD, and conclude that patients with PAD often have CAD disease.

There are some weak points that need to be addressed by the authors.

Major
1. In all patient analysis, the CAD patients showed a significantly higher prevalence of DM as compared with non-CAD subjects (P < 0.01). However, in age-matched analysis, there was no significant difference between two groups. The authors explain it.
**Response: Thank you so much for your careful review and important comment. In this study, the CAD group had a higher prevalence of DM than the non-CAD. DM, like CAD, is an important and independent factor affecting the survival of PAD patients. The propensity score matching used in this study is a method of adjusting all the possible baseline confounding variables such as DM, hypertension, smoking, alcohol drinking, laboratory findings, and others that can affect not only the age but also the endpoints of this study.**

2. As shown in Table 4, in all patients, total death (five-year outcomes) was higher in CAD group than in non-CAD group without significant difference. In contrast, in age matched analysis, it was higher non-CAD group than that of CAD patients. Why?

**Response: Thank you so much for your careful review and important comment. In all patient analysis of this study, the CAD group had more elderly, diabetic, and higher creatinine levels than patients in the non-CAD group (Table 1). Although not statistically significant, this baseline difference can explain the higher mortality rate in the CAD group. After PSM analysis (This is not an age matching method), although there was no statistical significance, in contrast, the non-CAD group had a high mortality rate. This is because non-cardiac death was higher in the non-CAD group than in the CAD group. As in "Matched patients" in Tables 1 to 3, the baseline characteristics of the two groups were well balanced by the PSM method, but the variables that could not be evaluated may have affected non-cardiac death. This is described in the limitations of the discussion part (line 7 on page 10 to line 9) as follows as “we could not adjust for all limiting factors not shown in medical records or collected through telephone contact.”**

3. Discussion sections were disorganized, the authors did not clearly figured out their own findings and did not derive clinical implication in each section based on your own and previous observations.

**Response: Thank you so much for your careful review and important comment. We actively revised the discussion to reflect your opinion. We attach a revised manuscript.**
