# Supplementary material for: Long-term outcomes of peripheral arterial disease patients with significant coronary artery disease undergoing percutaneous coronary intervention
Source: PLoS One. 2021 May 19;16(5):e0251542. doi: 10.1371/journal.pone.0251542 (PMC8133421; doi:10.1371/journal.pone.0251542)
Supplement: Attachment — Submitted filename: Pone-PTA-CAD-Rebuttal_R2.doc [file pone.0251542.s003.doc]

**Dear reviewer:**

**Thank you so much for your very insightful and serious comments for our manuscript! We really appreciate your excellent advice which has helped us a lot in improving our manuscript. According to your comments, we have amended the relevant parts in the manuscript. Some of your questions were answered below.**

**Yours sincerely,**

***Correspondence to:**

Seung-Woon Rha, MD, PhD, E‐mail: swrha617@yahoo.co.kr (S-WR)

**Reviewer #1:** The main finding of this study is that routine coronary angiography and subsequent percutaneous intervention for significant coronary artery disease in patients with symptomatic peripheral arterial disease is safe and resulted in similar long-term survival to those who did not have coronary artery disease.

1 The correlation of coronary and peripheral arterial disease is well known and treatment strategies have been discussed and examined. What therefore is the novelty in this study of its conclusions

Response: Thank you so much for your careful review and important comment. As you understand, the treatment of PAD and CAD is very important for improving patient’s survival and quality of life. However, in patients undergoing PTA, routine evaluation of the presence of significant coronary artery disease may be controversial. In real world clinical practice, physicians who are performing endovascular intervention commonly only focusing on the extremity target lesion intervention without concerning of coronary artery disease evaluation and management, especially in particular specialties such as vascular surgeon or interventional radiologists.
**ARE THERE ANY DATA TO SUPPORT THIS STATEMENT. WE ARE IN FACT CONCERNED ABOUT THE PRESENCE OF CAD IN PAD PATIENTS IN OUR INSTITUTION AND ELSEWHERE.**

**Response: Thank you so much for your careful review and important comment. As you know that the patients with PAD have particularly a high mortality rate from cardiovascular events. The severe CAD has been reported in 54% to 69% of patients with PAD [1-4]. So, the use of cardio-protective drugs as antiplatelet therapy, statin, and ACE inhibitors in PAD helps improve survival, but the introduction of these guidelines is more than a decade behind CAD. Also, the underuse of cardio-protective medication in the PAD population in comparison to patients with CAD has been published previously. These are well explained in Teraa's review. We cited his review in the revised manuscript [5].**

**Therefore, we changed the following in the second paragraph of the discussion part (line 4 to line 8 on page 11) as follows as “ In real world clinical practice, physicians who are performing endovascular intervention commonly only focusing on the extremity target lesion intervention without concerning of CAD evaluation and management. Also, the use of cardio-protective drugs such as antiplatelet therapy, statin, and ACE inhibitors in PAD helps to improve survival, but the introduction of these guidelines is more than a decade behind CAD.”**

[References]

1. Faglia E, Dalla Paola L, Clerici G, Clerissi J, Graziani L, et al. (2005) Peripheral angioplasty as the first-choice revascularization procedure in diabetic patients with critical limb ischemia: prospective study of 993 consecutive patients hospitalized and followed between 1999 and 2003. Eur J Vasc Endovasc Surg 29: 620-627.

2. Lee MS, Rha SW, Han SK, Choi BG, Choi SY, et al. (2015) Clinical outcomes of patients with critical limb ischemia who undergo routine coronary angiography and subsequent percutaneous coronary intervention. J Invasive Cardiol 27: 213-217.

3. Nishijima A, Yamamoto N, Yoshida R, Hozawa K, Yanagibayashi S, et al. (2017) Coronary Artery Disease in Patients with Critical Limb Ischemia Undergoing Major Amputation or Not. Plast Reconstr Surg Glob Open 5: e1377.

4. Ryu HM, Kim JS, Ko YG, Hong MK, Jang Y, et al. (2012) Clinical outcomes of infrapopliteal angioplasty in patients with critical limb ischemia. Korean Circ J 42: 259-265.

5. Teraa M, Conte MS, Moll FL, Verhaar MC (2016) Critical Limb Ischemia: Current Trends and Future Directions. J Am Heart Assoc 5.

2 How does a single center study that performed angiography on each patients help
a. Would it not have been more meaningful to compare those with symptoms who did and did not undergo angiography
Response: Thank you so much for your careful review and important comment. Korea University Guro Hospital (KUGH), which conducted this study, is a university hospital for training, and is a large-scale center that performs many CAG, PCI, and PTAs every year. Data are collected by a trained research coordinator using a standardized case report form. And professional researchers analyze it consistently. It may be smaller than a multicenter study, but it can collect and analyze data more efficiently and accurately. Our study is a comparative study of the CAD group and the non-CAD group who received appropriate treatment through coronary artery evaluation in patients undergoing PTA. Therefore, the group without CAG was excluded. Main purpose of this study is to show whether the concomitant optimal revascularization of significant CAD regardless of patient’s symptom in PAD patients undergoing PTA could reduce the cardiovascular event risk compared with PAD patients without significant CAD. Selective CAD evaluation according to ischemic symptoms in PAD patients undergoing PTA is not a suitable and safe strategy because many of them are elderly, diabetics and limited ambulation to provoke ischemic chest pain or dyspnea due to diabetic foot wound. Thus, selective symptom driven CAD evaluation cannot be ideally accepted in context of ‘pan-atherosclerosis’ of PAD patients. We will consider a comparative study in symptomatic PAD patients between CAG and non-CAG groups in future studies.
**THE FAILURE TO COMPARE THOSE WHO DID WITH THOSE WHO DID NOT UNDERGO ANGIOGRAPHY LEAVES NO DEFINITIVE CONCLUSION**

**Response: Thank you so much for your important comment. We fully agree with your opinion. In the revised manuscript, we analyzed by adding 91 cases (as defined as the no-CAG group) who did not perform routine coronary angiography. Patients were divided into three groups; 1) routine CAG and a presence of CAD (CAD group: 413 patients), 2) routine CAG without CAD (non-CAD group: 261 patients), and 3) no-CAG group (91 patients). During the 5-year clinical follow-up, the routine CAG group was associated with reduced risk of myocardial infarction by 65.3% as compared to the no-CAG group. Also, there were no differences in the incidence of any clinical events between the CAD group and the non-CAD group except for the incidence of repeat percutaneous coronary intervention (PCI), which was higher in the CAD group than the non-CAD group. These are the results section of the revised manuscript (lines 19 to 19 lines on 8 pages, 15 to 20 lines on 9 pages), the discussion section (lines 22 to 24 lines on 11 pages) and the statistical analysis part (lines 19 to 21 on 7 page) in the method section.**

**Figure 1. Survival analysis of 5-year clinical outcomes by Kaplan–Meier curve and Cox-proportional hazards regression analysis.**

Figure A,C,E, and G show an Un-adjusted hazard ratio by Cox-proportional hazards regression analysis. Figure B,D,F, and H show an adjusted hazard ratio by Cox-proportional hazards regression analysis. Adjusted confounders are Wound, Sex, Age, Hypertension, Diabetes mellitus, Chronic Renal Insufficiency, Current of smoking , treated limb side, and treated lesion (femoral, Popliteal, and Below the knee artery). MACCE, major adverse cerebrovascular and cardiac events; CI, confidence interval; CAD, coronary artery disease; CAG, coronary angiogram.

3 What new conclusions were learned not seen in ref, 8 the authors 1-year experience with this group
Response: Thank you so much for your careful review and important comment. The results of our previous study, which observed the results for one year, the CAD group that received optimal treatment through routine coronary artery evaluation showed similar clinical results than the non-CAD group in CLI patients received PTA [8]. Similarly, in the present observation of long-term clinical results of 5-year in patients undergoing PTA, the clinical results of the CAD and non-CAD groups were similar. The strategy for CAD evaluation and treatment in PTA patients seems to be a safe and effective strategy not only for better short-term outcomes but also durable long-term outcomes. We wanted to show the durable long-term results from this routine CAD work up and management strategy in PAD patients undergoing PTA. We added this to the discussion part (line 20 on page 9 to line 14 on page 10).
**WHY WOULD ONE EXPECT THAT LONG-TERM OUTCOMES WOULD BE SIGNIFICANTLY WORSE THAN SHORT TERM. THIS IS WHY THERE NEEDS TO BE A NON-ANGIOGRAM GROUP TO SEE NOT ONLY OUTCOME BUT CROSSOVER**

**Response: Thank you so much for your important comment. We fully agree with your opinion. As mentioned earlier, we analyzed by adding A NON-ANGIOGRAM GROUP (the no-CAG group) and reflected it in the revised manuscript.**

4 Was a power calculation performed to determine if 160 matched patients could indeed detect a meaningful difference
Response: Thank you so much for your careful review and important comment. This study was designed as a prospective registration study and retrospective analysis was performed. Therefore, the sample size after PSM may not be sufficient, which may be a limitation of the study. We added this to the limitations of the discussion part (line 17 on page 10 to line 19) as following as “Second, In the PSM analysis, a total of 320 patients from 160 pairs in both groups were analyzed. This sample size may be insufficient to produce results. Our study is a registered observational study and may be a limitation of analysis.”
**PERHAPS THE AUTHORS COULD ADD A STATEMENT AS TO HOW MANY PATIENTS WOULD HAVE NEEDED TO BE STUDIED TO SHOW A DIFFERENCE RATHER THAN SAY MAY BE INSUFFICIENT.**

**Response: Thank you so much for your careful review and important comment. Already, a randomized clinical trial similar to ours is in progress, and relevant information is described in the discussion part (lines 12 to 17 lines on 12 pages) as follows as “Similar to our research, Chen and colleagues have registered the multi-center randomized controlled trial (NCT02169258) “Routine Coronary Catheterization in Low Extremity Artery Disease Undergoing Percutaneous Transluminal Angioplasty (PIROUETTE-PTA)” at ClinicalTrials.gov [18]. Estimated enrollment is 700 participants and the primary endpoint of the study was composite major adverse cardiac events at the 1-year follow-up.”**

**Also, we change this to the limitations part of the discussion section (line 17 to line 20 on page 12) as follows as “Second, In the PSM analysis, a total of 320 patients from 160 pairs in both groups were analyzed. This sample size may be insufficient to produce results. Our study is a registered observational study and may be a limitation of analysis. As Chen and colleagues' research results come out, the results will be updated a bit.”**
