# Supplementary material for: Long-term outcomes of peripheral arterial disease patients with significant coronary artery disease undergoing percutaneous coronary intervention
Source: PLoS One. 2021 May 19;16(5):e0251542. doi: 10.1371/journal.pone.0251542 (PMC8133421; doi:10.1371/journal.pone.0251542)
Supplement: Attachment — Submitted filename: R3 PTA-CAD-MS rebuttal.doc [file pone.0251542.s004.doc]

**Dear reviewer:**

**Thank you so much for your very insightful and serious comments for our manuscript! We really appreciate your excellent advice which has helped us a lot in improving our manuscript. According to your comments, we have amended the relevant parts in the manuscript. Some of your questions were answered below.**

**Yours sincerely,**

***Correspondence to:**

Seung-Woon Rha, MD, PhD, E‐mail: swrha617@yahoo.co.kr (S-WR)

Reviewer #1: I find this paper somewhat confusing still. The authors have tried to respond to my inquiries but as best as I can tell there is a barely statistically significant difference in groups that did and did not undergo angiography in the setting of symptomatic peripheral arterial disease.

This remains in my mind a not compelling and in fact a confusing story
I this remains confusing – there are three groups – two that had routine angiography one with and one without coronary artery disease and a third who did not undergo angiography. And the five year clinical follow up is that both groups who underwent coronary angiography were equivalent but had minor HR 0.347 p=0.049 reduction compared to those who did not. In other words regardless as to whether coronary artery disease was discovered and regardless as to whether an intervention was performed.
Is this correct?
If this is correct – what is the message that angiography is to be performed

Is it the case that there is benefit on the crossover group only or the entire group?

**We sincerely appreciate your comments. Your point is correct. Although the risk of MI in the CAG group (HR: 0.347, 95% CI: 0.121 to 0.998, p=0.049, Figure 1, F) was lower compared to the no CAG group, this is due to the no CAD group (HR: 0.204, 95% CI: 0.049 to 0.852). The risk of MI in the CAD group (HR: 0.523, 95% CI: 0.184 to 1.485) was lower than that of the group without CAG, but there was no statistical significance (This statistical values were not shwon on the table and figure). Therefore, this might be confusing to readers and reviewers. In the revised manuscript, all statements that the MI risk reduction due to the CAG test has been deleted and changed. Comparing 3 groups appears to be clinically important and relevant due to it is practically reflecting real world clinical practice. Some proportion of patients definitely refuse to undergo coronary angiography due to absence of symptoms, cost issues and other personal preferences. Thus we need to know the long term impact of the active coronary surveillance in advanced PAD patients undergoing PTA.**

II Moreover, the text is till confusing – take for example the next to last paragraph which reads

Third, all subjects in this study underwent CAG, and therefore our results are not generalizable to patients who do not receive CAG. Finally, all the PAD patients with significant CAD patients did not undergone PCI due to some reasons including clinical judgment based upon the discretion of the clinician and patient preference. Only 71.6% of all CAD patients underwent PCI or CABG before, after or at the same time as admission for PTA. This registry reflects the real-world practice of PAD patients.

1 the text is unclear – there are some extra words
2 was the benefit referred to in all patients who had CAG irrespective of whether they
a. had CAD
b. had an intervention

or were only the 71.6% of all CAD patients who underwent PCI or CABG considered?

**We sincerely appreciate your comments. We have made some changes to this statement in the limitation section as follows as “**The results of our study were derived indirectly by comparison with the survival rates of other studies. In general, the 5-year survival rate of PAD patients is around 50% [15], and the survival rate of our study subjects, 88.5%, was very high. The no CAG group without cardiac function evaluation should be allocated to verify the effectiveness of routine CAG in PAD patients, but this is a problem that may be against research ethics. In this study, PTA was performed by an interventionist based on cardiology, the cardiac function of all subjects was evaluated non-invasively and/or invasively by the cardiologist. Therefore, the no CAG group in this study should not be confused with the patients that did not perform cardiac function evaluation.**” Also, we have some changes in the method section as follows as "**A total of 765 consecutive PAD patients underwent successful PTA and 674 patients underwent routine CAG. For the remaining 91 patients who did not receive the CAG, but the cardiac function was evaluated non-invasively by the cardiologist. **"**

**This latter issue is further confusing by the note on page “In the present study, the CAG group had reduced significantly the risk of myocardial infarction by 65.3% than the no-CAG group during 5 years of clinical follow-up.”
We sincerely appreciate your comments. Your point is correct. Although the risk of MI in the CAG group (HR: 0.347, 95% CI: 0.121 to 0.998) is lower compared to the no CAG group, this is due to the no CAD group (HR: 0.204, 95% CI: 0.049 to 0.852). The risk of MI in the CAD group (HR: 0.523, 95% CI: 0.184 to 0.1.485) was lower than that of the group without CAG, but there was no statistical significance. Therefore, in the revised manuscript, all statements that the MI risk reduction due to the CAG test has been deleted and changed. The main finding of this study is that routine CAG and subsequent PCI for significant CAD in symptomatic PAD patients undergoing PTA is safe and resulted in similar long-term survival as compared with the symptomatic PAD patients who undergoing PTA and who did not have CAD. Not surprisingly, repeat PCI was performed more frequently in PAD patients with CAD at long-term follow-up. We wanted to demonstrate the risk reduction of advanced PAD patients with significant CAD patients by providing long-term clinical benefit from active coronary revascularization as compared to those of PAD patients without significant CAD. Thus, wanted to show the clinical benefit of active concomitant coronary surveillance in advanced PAD patients undergoing PTA to improve their prognosis.**

III the idea that this paper is a follow up to reference 8 is confusing given that the numbers are so different

**We sincerely appreciate your comments. We agree with your comments. This study differs from the study in Reference 8 in its study subjects and study duration. Therefore, the statement of reference 8 has been deleted and changed in the revised manuscript.**
